# Inferring a causal relationship between ceramide levels and COVID-19 respiratory distress

**DOI:** 10.1038/s41598-021-00286-7

**Published:** 2021-10-21

**Authors:** Mehran M. Khodadoust

**Affiliations:** Vexo Pharmaceuticals, DMCC, Dubai, UAE

**Keywords:** Biomarkers, Drug development, Translational research, Viral infection

## Abstract

A causal relationship between plasma ceramide concentration and respiratory distress symptoms in COVID-19 patients is inferred. In this study, plasma samples of 52 individuals infected with COVID-19 were utilized in a lipidomic analysis. Lipids belonging to the ceramide class exhibited a 400-fold increase in total plasma concentration in infected patients. Further analysis led to the demonstration of concentration dependency for severe COVID-19 respiratory symptoms in a subclass of ceramides. The subclasses Cer(d18:0/24:1), Cer(d18:1/24:1), and Cer(d18:1/22:0) were shown to be increased by 48-, 40-, and 33-fold, respectively, in infected plasma samples and to 116-, 91- and 50-fold, respectively, in plasma samples with respiratory distress. Hence, monitoring plasma ceramide concentration, can be a valuable tool for measuring effects of therapies on COVID-19 respiratory distress patients.

## Introduction

The COVID-19 virus penetrates vital organs such as the lung with a range from asymptomatic or mild infections restricted to the upper respiratory tract to severe respiratory syndromes hallmarked by disseminated spread to the lower airways. Observation of local inflammation and pneumonia, especially in patients with preconditions such as diabetes, hypertension, and cardiovascular disease, has also been reported^1^. Supportive supplemental therapy for alleviation of these respiratory distress symptoms is the most viable strategy and the goal of most current efforts for improving COVID-19 patient survival.

Recent studies have highlighted that after infection, some viruses can highjack and utilize host lipid metabolism for their own propagation and to counteract cellular responses^2,3^. To explore evidence for similar virus host exchanges and to better understand the mechanisms by which COVID-19 infects host cells, in addition to this study a number of metabolomics studies have been reported^4–12^.

Schwarz et al. described the progression from moderate to severe disease to be marked by loss of specific immune regulatory lipid mediators (LMs) and increased proinflammatory constituents^5^. Similarly, Wu et al. reported that in addition to dyslipidemia, major changes to carbamoyl phosphate and guanosine monophosphate (GMP) levels are also associated with the progression and severity of COVID-19^6^. Shen et al., in a metabolomics and proteomic study, revealed characteristic protein and metabolite changes in the sera of severe COVID-19 patients, which might be used in the selection of potential biomarkers for the evaluation of the severity of disease^7^. Significantly altered lipid metabolism was also reported by Thomas et al., in particular, short- and medium-chain saturated fatty acids, acyl-carnitines, and sphingolipids in RBCs from COVID-19 patients^8^. All these studies have reported unbiased analyses of patient plasma samples, and all have shown that changes in the Lipidome are associated with severe COVID-19 infection. Ceramides (Cer) are the products of the metabolism of fats and lipids. They have been reported to accumulate in humans with obesity and hyperlipidemia. They are the central metabolites of the sphingolipid family and have also been reported to be signaling molecules that can regulate ER stress, apoptosis, insulin sensitivity and inflammation^13,14^. Increased Cer levels are heavily implicated in the pathogenesis of insulin resistance, neurodegeneration conditions, and lung diseases, including asthma, chronic obstructive pulmonary disease (POD), and pulmonary fibrosis^15^.

The aim of the present study was to obtain metabolomics data from three cohorts, that is COVID-19 uninfected, infected with mild symptoms, and infected with severe symptoms in a statistically meaningful manner for the assembly of an observational constraint-based approach that could be used for inference of causal discovery. The typical approach for identifying causal relationships between potential metabolic signals and the factors they regulate, is by specifically designed experiments for the generation of dynamic multi-omics datasets that systematically incorporates prior knowledge. In such studies if a metabolite associated with the risk factor are also shown to be associated with the outcome, then the plausibility that the risk factor is a causal determinant of the outcome is increased. Observational data can be used to constrain the causal relationships among measured variables, sometimes to the point that one can infer that a variable is causing another variable^16,17^.

## Results

### Identification of differential metabolites

The ions corresponding to d7-androstenedione, d4-cortisol, and d5-DHEAS, used as internal standards, were selected for extraction of the ion chromatographic peaks in an LC/MS/MS-based comparative metabolomics analysis.

**Figure 1:**
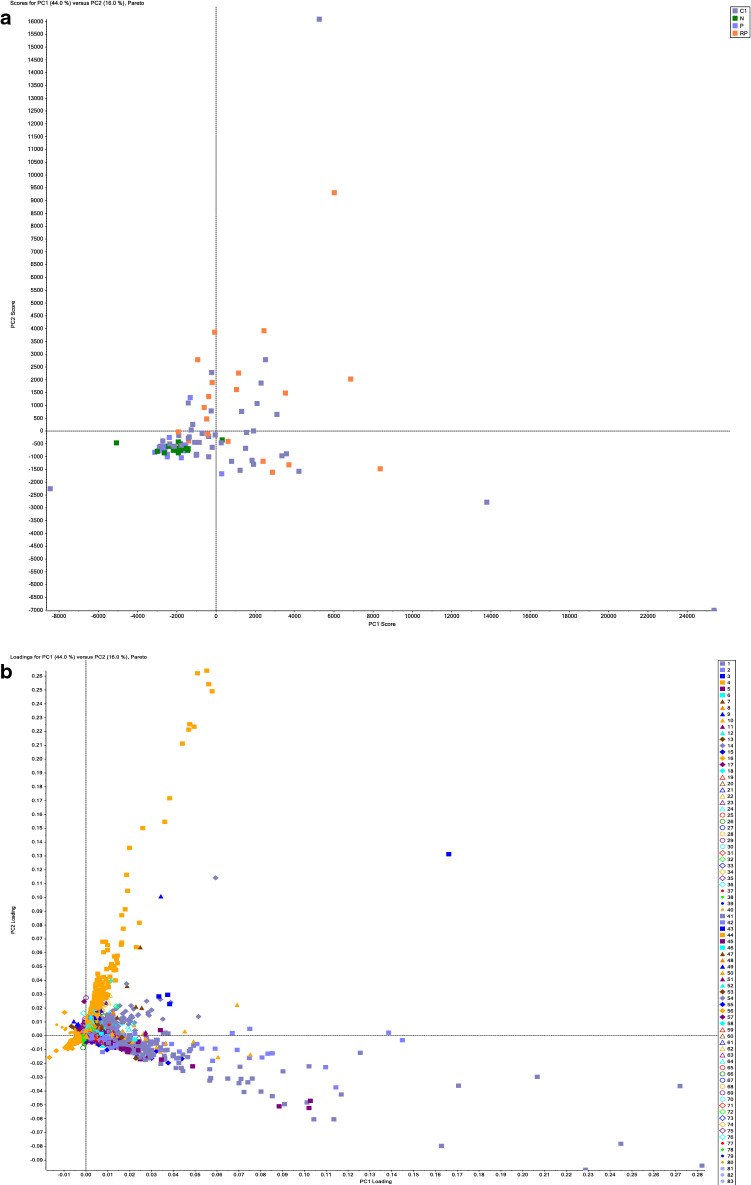

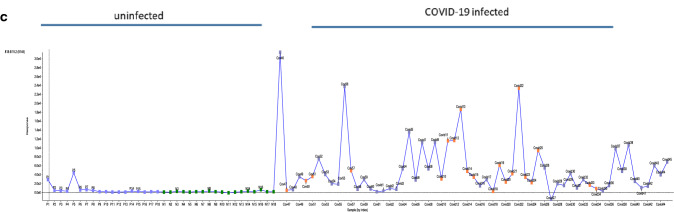
Metabolomics analysis of plasma samples. (**a**) Scoring plots from PCA model analysis of the full-scan MS analysis for classifying COVID 19 infected (dark purple and orange squares) and control groups (light purple squares: uninfected male, green square: uninfected female). and (**b**) loading plots from PCA model analysis in which each metabolite is represented by a distinct color as shown in the graph legend (**c**) Profile of an identified regulated metabolite, via loading component analysis, as represented by peak area across COVID-19-infected and control samples, such metabolites drives the observed separation seen between the cohorts in a PCA model analysis, using MarkerView software by SCIEX. COVID 19 infected (dark purple and orange squares) and control groups (light purple squares: uninfected male, green square: uninfected female).

Figure 1a shows the obtained score plots from metabolomics profiles of plasma from the COVID-19-infected and control uninfected groups. The clear separation shows that significantly different metabolites are present within each group. The loading plots shown in Fig. 1b demonstrate these differences at the component level. The ions that are furthest from the origin of the plots are the ones that make the most contribution to the separation seen in the score plots and were selected for further analysis. Figure 1c shows a profile of a metabolite chosen from the loading plot.

Each component of the loading plot was investigated, and a list composed of the m/z of selected ions with a define profile of abundance and characteristics was formed. Accurate molecular ion masses were used for composition, calculation, and collection of data-dependent MS/MS data. MS/MS data were collected for structural deduction and database searches.

### Identification and structural deduction of metabolites via data-dependent MS/MS

**Figure 2:**
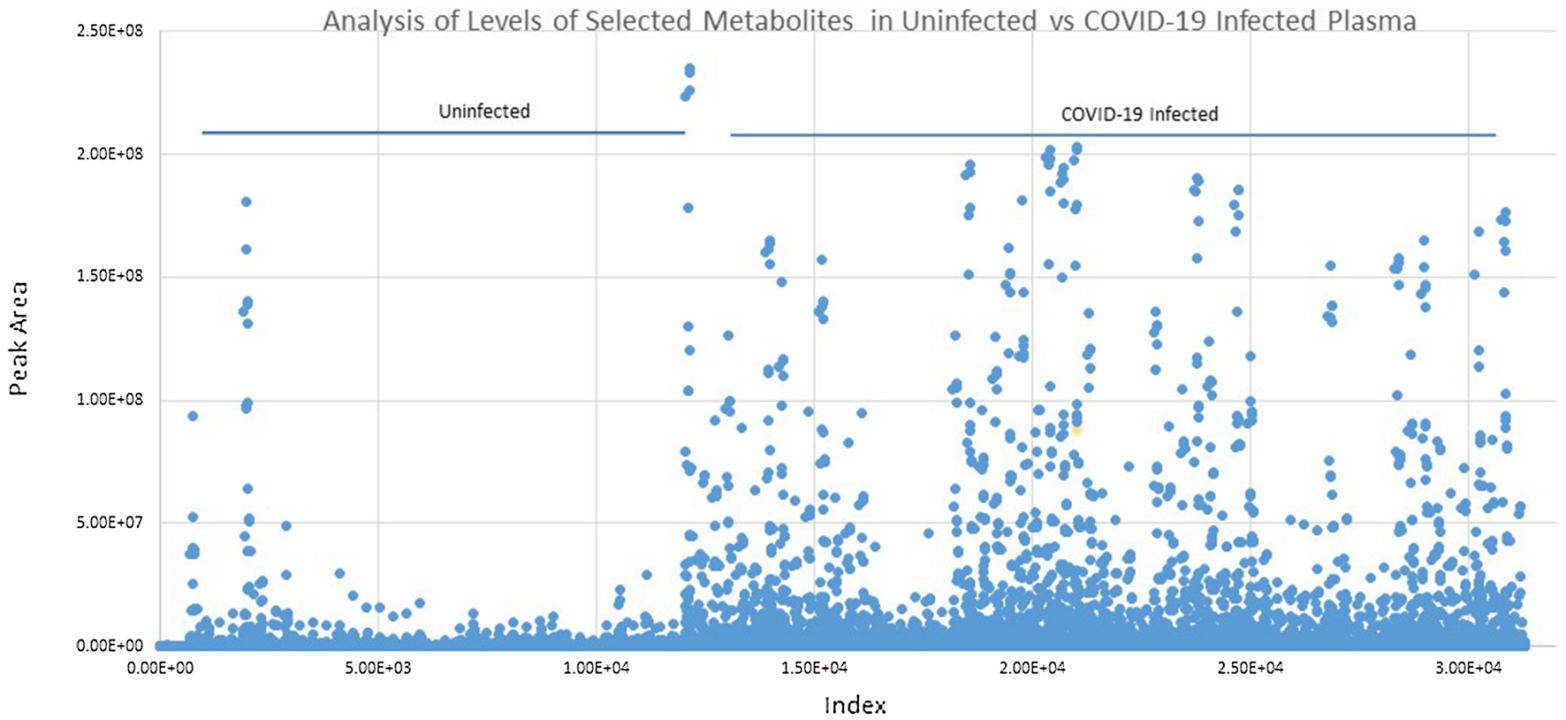
COVID-19 infection and abundance of selected metabolites. A comprehensive over view of levels, as measured by peak area, of metabolites in COVID-19-infected and control samples as measured by LC–MS/MS IDA analysis utilizing the m/z inclusion list provided from the loading component of PCA analysis. Each spot represents a metabolite as defined in the inclusion list, the Y axis represents the peak area detected for each m/z ion across the analyzed uninfected and COVID-19-infected samples X- axis.

Figure 2 shows an overview of the results from the analysis of plasma from uninfected and COVID-19-infected individuals using a data-dependent MS/MS method. The combined monitored m/z ion profile from this analysis confirms the principal component analysis (PCA)-directed selection process. Structural deduction and database searches demonstrate that > 283 of the metabolites identified belong to the lipid family.

### From metabolomics to lipidomics

**Figure 3:**
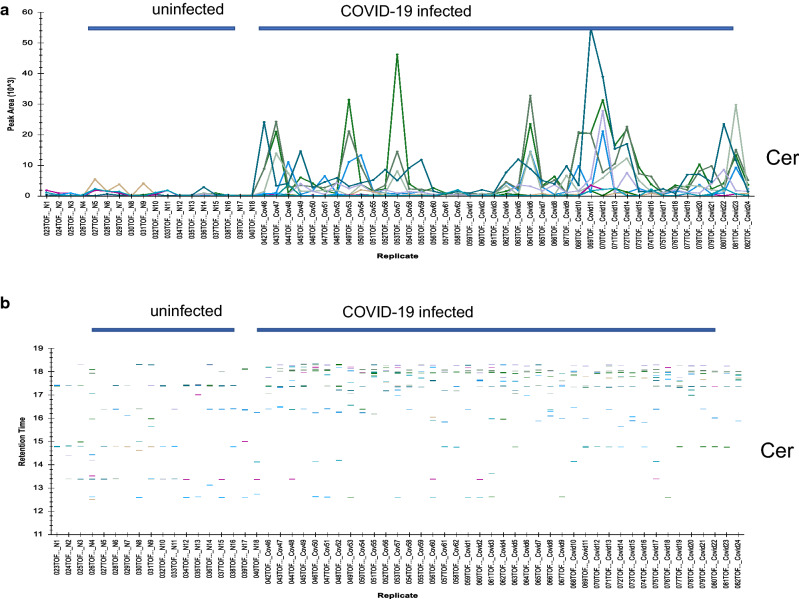

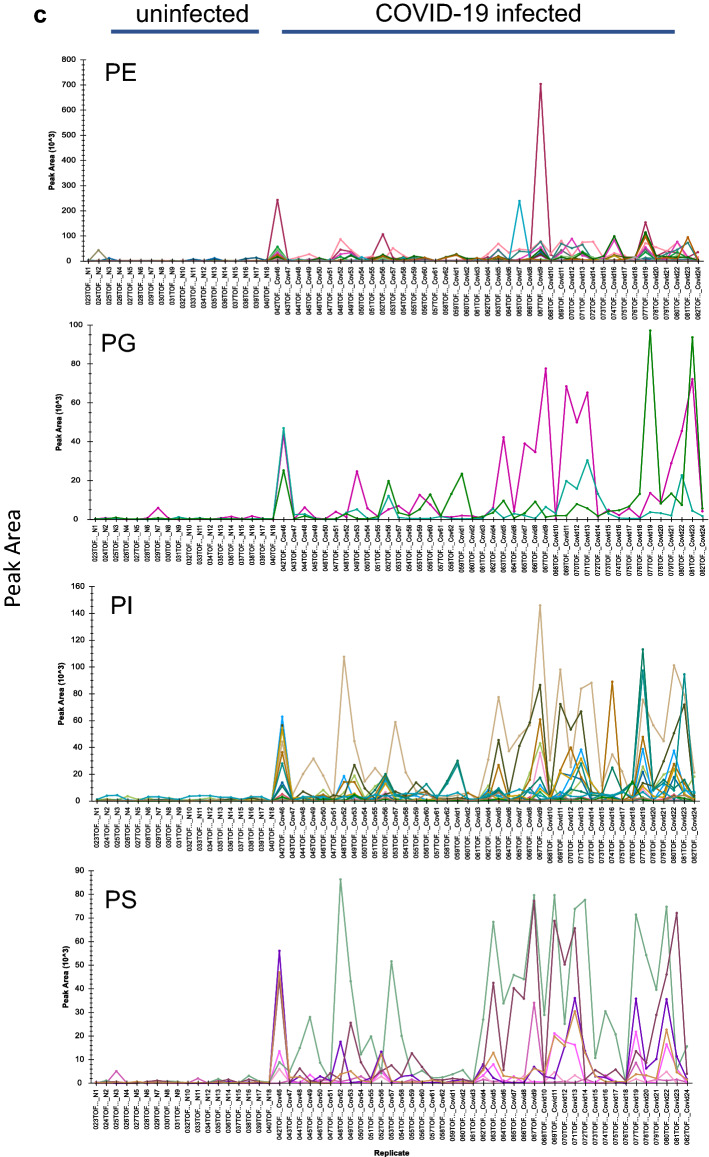
Grouping of identified COVID-19 infection-dependent metabolites into lipid classes. Comparative abundance profile of the identified subclasses of lipids per class in the plasma of uninfected and COVID-19-infected individuals. An over view of levels, as measured by peak area (Y- axis), of metabolites in COVID-19-infected and control samples as measured by LC–MS/MS IDA analysis. Each color line represents a metabolite as defined in the inclusion list, grouped into lipid subclass, and lipid class. (**a**) Analysis of observed changes in peak area of subclasses of the Cer (Y-axis) in uninfected (samples N1–N18) and COVID-19-infected plasma samples (X-axis, Samples Covid1–24, Covid 46–62). (**b**) Changes in the observed retention time of the subclasses of Cer across all samples. (**c**) Analysis of observed changes in peak area of subclasses of the PE, PG, PI, and PS class (Y-axis) in uninfected and COVID-19-infected plasma samples (X-axis). Each metabolite defined in the inclusion list is represented in a different color.

Figure 3a shows the identification, categorization and quantification of identified lipid metabolites: PS = Phosphatidylserine, Cer = Ceramides, PE = Phosphatidylethanolamine, PI = Phosphatidylinositol, PG = Prostaglandin, HexCer = Hexosylceramide, Hex2Cer = DiHexosylceramide, Hex3Cer = TriHexosylceramide by using the combination of obtained peak list of selective ions, obtained MS/MS data, and LIPID CREATOR platform^18^. Figure 3b shows the observed retention time (RT) differences between the identified metabolites in the plasma of uninfected and COVID-19-infected individuals. A molecular weight or isomerization change of lipid subclasses can be seen by changes in its RT. Hence, the presence of new Lipid subclasses can be detected by comparing the RT of subclasses of the lipids in each class.

**Figure 4:**
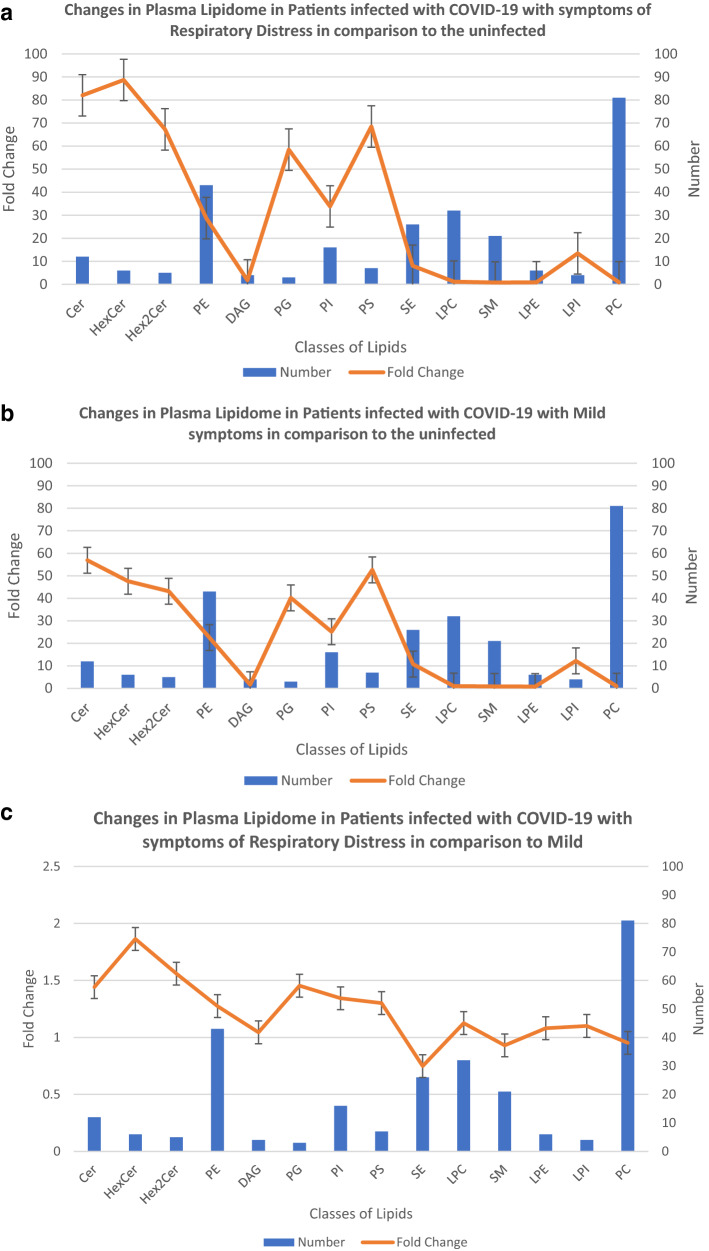
Effect of Covid-19 infection on Plasma Lipidome. Metabolites identified to be different lipid subclasses are segregated based on their class (X-axis), the number of members of each class identified in the plasma of COVID-19 infected individuals is represented (Y-axis). The fold sum of MEDM changes in peak areas, shows average change in levels of the identified lipid class as you go from the uninfected to infected with COVID-19 with respiratory distress symptoms (**a**), and to infected with mild symptoms (**b**). The comparative differences between the lipid classes in plasma of individuals with COVID-19 infection with mild vs respiratory distress symptoms are monitored (**c**). Error bars represent, standard deviation of replicates.

The changes in levels of each lipid class can be represented by showing the average fold sum of median of means (MEDM) changes in the peak area of each identified lipid subclass in that class relative to the uninfected. Figure 4a shows the changes in the number of lipid subclasses observed in the plasma due to COVID-19 infection with mild symptoms compared to the uninfected candidates, in addition to the average fold sum of MEDM changes per class. Figure 4b also shows the changes in the number of subclasses of lipids per class in the plasma of individuals infected with COVID-19 with respiratory distress symptoms in comparison to the uninfected group, in addition to the average fold sum of MEDM changes per lipid class. Figure 4c demonstrates the changes in the average fold sum of MEDM and the number of subclasses of each of the lipid classes in the plasma of the patients with COVID-19 infected with mild symptoms vs infected with respiratory distress symptoms.

In the plasma samples from COVID-19 patients with mild symptoms (Fig. 4a) and patients with respiratory distress symptoms (Fig. 4b), an average 40-fold increase in the concentration of lipid classes Cer, HexCer, Hex2Cer, Hex3Cer, PG, and PS was observed. Figure 4c shows that when only comparing the metabolic differences in the plasma of the Covid-19 infected with mild to the severe symptoms, the observed changes in lipid profiles become limited to Cer (average more than 1.5–2 fold increase this study) and its derivatives HexCer, Hex2Cer.

### Covid-19 respiratory distress and Cer

**Figure 5:**
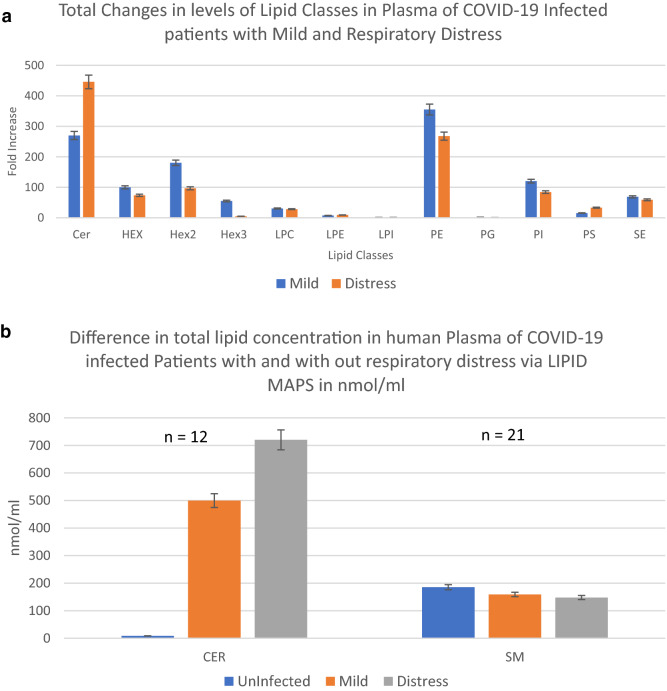
Total Levels of Ceramide class of lipids in Plasma of the Covid-19 infected. Fold sum of MEDM changes of peak area times number of subclasses identified per lipid class. (**a**) An overall estimation of relative changes in the concentrations of various lipid classes. (**b**) Relative concentration of lipids per class in μmoles/l of plasma as determined via the SRM 1950 consensus LIPID MAPS consortium. Error bars represent, standard deviation of replicates.

The compositional makeup of the subclasses of lipids present in the plasma of COVID-19-infected patients with mild symptoms vs those with respiratory distress shows no change at the subclass levels in each class of the identified lipids (Supplementary Figure 1). The overall change in plasma concentration of each lipid class is estimated by multiplying the average fold increases in peak area of each subclass by the number of identified lipid subclasses. Figure 5a demonstrates the concentration of each lipid class in relative terms of fold induction to the uninfected control group. Figure 5b shows the calculated plasma concentration of Cer and Sphingomyelin (SM) class of lipids in µmol/l in the uninfected, infected with mild, and infected with severe respiratory distress symptoms. SM, which is not modulated in plasma by COVID-19 infection (Fig. 4a–c), serves as a control in this study. These figures show that the total levels of Cer are increased in the plasma of COVID-19-infected individuals with mild symptoms by more than 250-fold or 500 µmol/l. In cases of COVID-19-infected individuals with severe respiratory distress symptoms, Cer levels were increased by over 450-fold or 720 μmol/l (Fig. 5a,b). In summary, an increase in 220 µmol/l plasma Cer concentration is the change seen between a COVID-19 infection with mild symptoms and an infection with a severe respiratory distress response.

**Figure 6:**
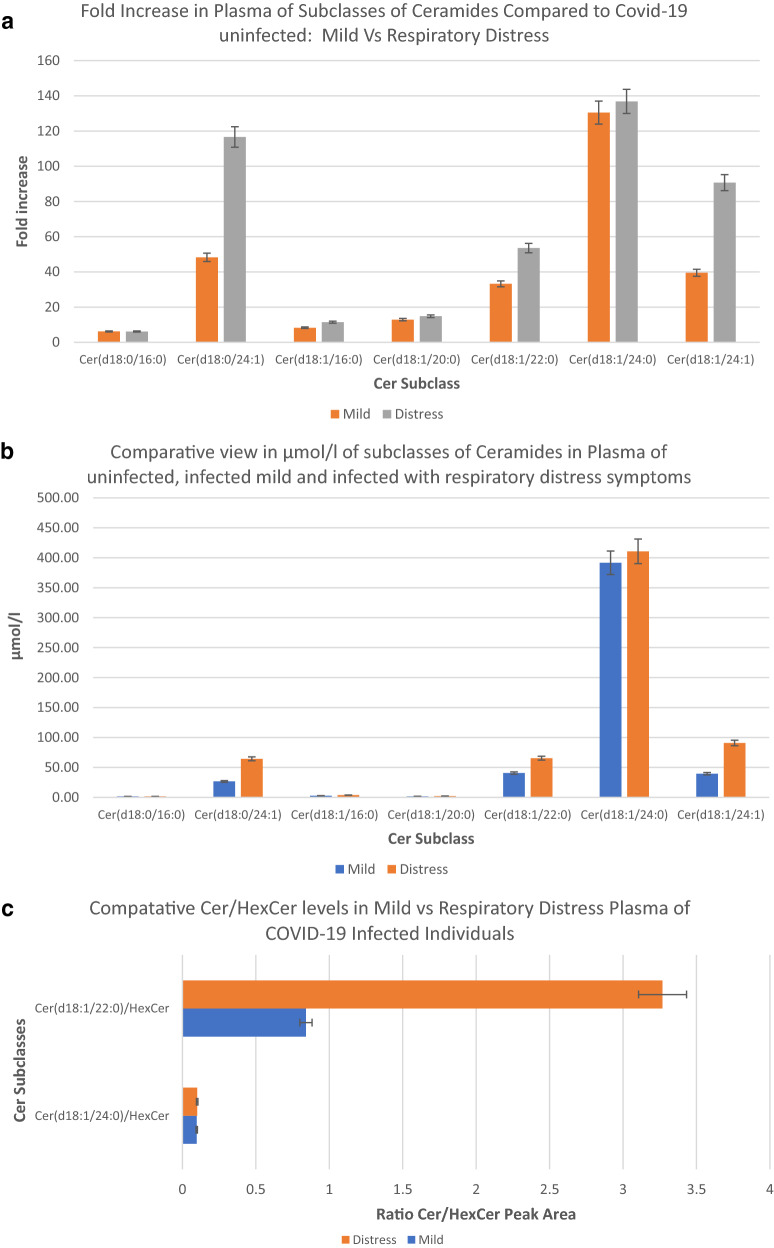

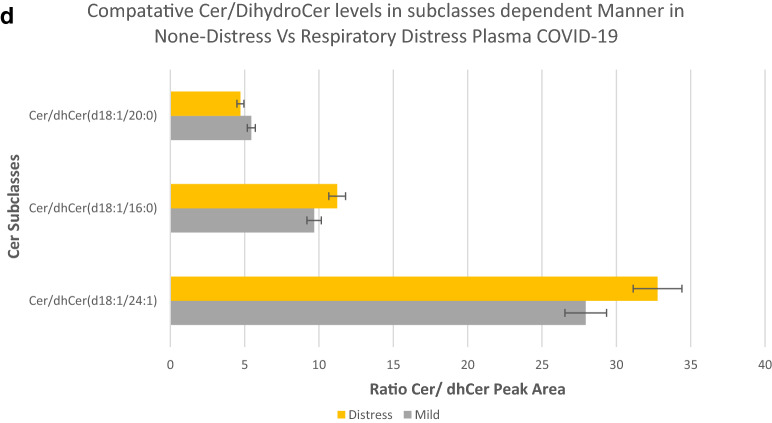
Ratios of lipids as predictive tests for respiratory distress. Abundance profile of subclasses of ceramides in the plasma of COVID-19 infected patients with mild and respiratory distress symptoms in terms of fold changes in observed peak area, compared to the plasma of uninfected individuals (**a**), Comparative view of changes in concentration of ceramide subclasses in the plasma of COVID-19-infected mild and with respiratory distress symptoms, from the uninfected, in terms of µmol/l (**b**), Relative abundance profile of subclasses of ceramides as represented by the ratio of peak areas of Cer/HexCer (**c**) and Cer/dhCer (Ceramide / dihydroceramide) ratios (**d**) in the plasma of COVID-19 infected patients with mild vs respiratory distress symptoms. Error bars represent, standard deviation of replicates.

Figure 6a shows breaking down the increases in plasma Cer concentration back into subclass levels. This figure shows the relative contribution of each of the Cer subclasses in terms of fold increases in plasma concentration compared to the uninfected and mild vs respiratory distress patients. This figure shows a range of 20- to 160-fold increases relative to the uninfected group in the concentrations of Cer(d18:0/24:1), Cer(d18:1/24:1), Cer(d18.0/20:0), and Cer(d18:1/20:0) in the plasma of mild and respiratory distress patients. Figure 6b shows a comparative measurement of the concentration of the Cer subclasses in plasma in μmol/l, as related to Covid-19 infection (SRM 1950—metabolites by LIPID MAPS consortium was the basis for comparison and establishment of concentration^19^). The overall levels of Cer(d18:1/16:0) were 2.79 μmol/l, Cer(d18:1/24:1) was 39.57 μmol/l in the plasma of COVID-19-infected individuals with no symptoms of distress, Cer(d18:1/16:0) was increased to 3.63 μmol/l (30% increase), and Cer(d18:1/24:1) was increased to 95.63 μmol/l (142% increase) in the plasma of respiratory distress individuals.

Ratios of lipids can be a very informative tool for the process of identifying more selective targets for the regulation of each Cer subclass. Figure 6c,d show comparative ratios of the identified subclasses Cer with their associated dihydroceramide and monohexosylceramide species in the plasma of COVID-19-infected individuals with mild vs severe respiratory distress symptoms. These figures demonstrate both the presence and relative ratio of the Cer substrates to Cer in a subclass-dependent manner. Figure 6d demonstrates the relative abundance of substrates for de novo synthesis of Cer(d18:1/20:0), Cer(d18:1/16:0), and Cer(d18:1/24:1) in the plasma of COVID-19-infected individuals.

## Discussion

A causal relationship between plasma levels of Cer and the onset of COVID-19 respiratory distress symptoms is inferred. Cer involvement has been highlighted in reports of lung diseases, including acute lung injury, cystic fibrosis, emphysema, lung infections, and asthma^13,15^. These reports show pulmonary manifestations to be the main symptoms of Cer-mediated toxicity. Cers can regulate major aspects of lung endothelial cell function and are involved in the pathogenesis of several conditions associated with pulmonary vascular dysfunction^15^. These pulmonary manifestation symptoms are common with the symptoms also seen in patients with COVID-19-associated respiratory distress^20^. The reported high levels of plasma Cer concentration in obese and asthmatic individuals are consistent with the observed sensitivity of this group to COVID-19 infection, supporting its biological relevance^21^.

Here we demonstrate both association, and concentration dependency of specific subclasses of Cer, in the plasma of COVID-19 infected individuals, with the observed respiratory distress symptoms. Causality is inferred, when constraints of biological relevance, previous metabolic studies reporting association, are incorporated with these acquired observational data.

COVID-19 infection causes profound changes in the metabolome of infected individuals, including plasma lipid levels. In this study, changes in plasma levels of 283 lipids covering 8 lipid classes, including PS, PE, PG, Cer, HexCer, Hex2Cer, and Hex3Cer, were identified to be associated with COVID-19 infection. Figure 3a–c shows the levels of subclasses of the above classes, as represented by peak area, to be markedly increased in the plasma of COVID-19 infected individuals, describing an association between the identified subclasses of lipids and COVID-19-infected plasma. This is consistent with the previous reported metabolomics studies^4–12^.

Figure 5a shows changes in the concentration of Cer as a class. In terms of fold induction, the total levels of Cer were increased in the plasma of COVID-19-infected individuals with mild symptoms by more than 250-fold or to 500 µmol/l (Fig. 5b). In cases of COVID-19-infected individuals with severe respiratory distress symptoms, this total Cer level is increased by over 450-fold or to 720 µmol/l, demonstrating concentration dependency. In other words, the difference between a COVID-19 infection with mild symptoms and an infection with severe respiratory distress response at the lipid class level is an increase of 220 µmol/l (or 44%) in plasma of total Cer class concentration. The predominant Cer subclasses (Fig. 6a, b) detected to be associated with these plasma levels in respiratory distress COVID-19 infection were Cer(d18:1/16:0) at 3.63 μmol/l and Cer(d18:1/24:1) at 95.63 μmol/l. The concentrations were 2.79 µmol/l (30% less) and 39.57 µmol/l (142% less), respectively, in the plasma of COVID-19-infected individuals with no symptoms of distress.

In Fig. 6c, the almost 2-fold greater Cer(18:1/22:0) to its hexosyl derivative ratio in respiratory distress patients shows a tool to predict severe respiratory distress and a possible mechanism for modulation.

The Cer(d18:1/16:0) and Cer(d18:1/24:1) subclasses were reported to be involved in a cardiac mortality study and to be associated with an increased risk of cardiac death outcomes in patients with stable coronary artery disease^22^. In this study, we report that COVID-19 infection causes a significantly greater mortality risk with a 10- and 30-fold increase in plasma levels of these subclasses over the reported cardiac mortality prediction level.

Causality prediction leads us to an advanced reasoning that monitoring and therapeutic aims of reducing Cer(d18:1/16:0) and Cer(d18:1/24:1) levels in the plasma of patients could enhance survival from COVID-19 respiratory distress. Hence, a reduction in plasma levels of the above Cer subclasses (bringing down the observed plasma levels to the level seen associated with mild symptoms) can be an aid in the recovery process of the severe symptoms associated with Covid-19 infection. This prediction can also shed light on new diagnostic and monitoring approaches for which further research is needed.

Cer is generated by de novo synthesis, salvage of sphingosine, and breakdown of complex sphingolipids, including sphingomyelin. The necessary substrates are monohexocylceramide, sphingomyelin, and dihydroceramide^23^. An increase in all three of these substrates was observed in the plasma of COVID-19-infected individuals (Figs. 3a, 6a,b).

Future studies should address more effectively the limitations of this study, the third criterion for causality, which requires alternative explanations for the observed relationship between two variables to be ruled out (nonspuriousness, or “not false.”). To better address these criteria, one would need to directly monitor the effect of lowering plasma concentrations of Cer by treatment with Cer inhibitors on COVID-19-mediated respiratory distress. To effectively modulate the toxic effects of Cer in the context of COVID-19 infection is to reduce its circulating concentrations. This can be achieved by inhibiting its biosynthesis or by chemical modification of the circulating Cer in the plasma. The Cer de novo synthesis pathway includes a series of enzymes that produce Cer from the starting components serine and palmitoyl CoA. Researchers seeking to pharmacologically inhibit Cer synthesis in vivo have generally used myriocin, which inhibits serine palmitoyltransferase, the rate limiting initial step in the biosynthesis of all sphingolipids^23^.

Ceramide synthase (CerS) catalyzes the acylation of the amino group of sphingosine, sphinganine, and other sphingoid bases using acyl CoA esters. CerSs consist of six enzymes (CerS1–6), with each isoform synthesizing a subset of Cer with partially distinct acyl chain lengths. CerS1 forms Cer18, CerS2 forms Cer24, and CerS6 forms Cer16 ceramide^24^. Since Cer(d18:1/16:0) and Cer(d18:1/24:1) are the predominant subclasses seen in the plasma samples of the COVID-19-infected group, focusing on modulation of CerS2 and CerS6 seems to be the logical therapeutic approach. Inhibitors of the Cers2 enzyme include fumonisins, the related AAL toxin, and australifungins^25^.

A reduction in the circulating concentration of Cer in the plasma can be achieved by causing chemical modifications with the introduction of enzymes or agonists to at least one ceramide-modifying enzyme, such as glucosylceramide synthase, ceramidase (SMase), ceramide kinase, and sphingomyelin synthase. Both acid and neutral SMase inhibitors, as well as inhibitors of the de novo pathway of Cer synthesis, effectively inhibited Cer-induced apoptosis in the lung in various acute or chronic injury models in vivo, as recently reviewed by Uhlig and Gulbins^25^. Carpinteiro et al. reported that pharmacological and genetic inhibition of the acid SMase enzyme can prevent infection of cells with COVID-19, VSV and PP. VSV viruses^24^.

Indirect examples of regulators or circulating Cer levels have also been described to include TNF-alpha inhibitors, TLR-4 inhibitors, adiponectin, FGF21, apoptosis inhibitors, and mitophagy inhibitors^26–28^.

Furthermore, our investigations have focused on the detected subclasses of Cer isolated through a non-comprehensive metabolomics analysis paradigm, and a more comprehensive approach may lead to the identification of more sensitive subclasses of these lipids.

In summary, uncovering causal relationships is an important first step towards understanding disease and predicting the course of future treatments. In this study, a causal relationship between plasma Cer plasma concentration and respiratory distress symptoms in COVID-19 patients is inferred. Specific subclasses of Cer have been identified that can be used for monitoring COVID-19 infection severity and progression. Since a causality relationship also defines that modulating ceramide synthesis pathways, its salvage and its regulatory mechanisms to be validated approach towards enhancing survival from COVID-19 respiratory distress further investigation on this prediction is warranted.

## Materials and methods

### Materials

Ammonium formate and formic acid were purchased from Sigma Aldrich Chemicals Co. (St. Louis, Mo, USA), LC–MS Grade Methanol, and Optima-grad acetonitrile (ACN) from Fisher Scientific (Pittsburg, PA, USA). A Millipore Milli-Q purification system (Bedford, MA, USA) was used to prepare deionized water. A total of 50 human plasma samples (K3EDTA, 37 female and 14 male) from active COVID-19-infected participants (positive via PCR), including 18 with severe respiratory distress and 32 with mild symptoms, were purchased from PRECISION FOR MEDICINE (Norton, MA, USA). All biospecimens have been certified to have been collected under a clinical study that has been reviewed by an institutional/Independent Review Board (IRB) in accordance with the requirements of local governing regulatory agencies, including the DHHS and FDA Codes of Federal Regulations, on the Protection of Human Subjects (45 CFR Part 46 and 21 CFR Part 56, respectively). The participants’ age range was 19–83 years, with 37 of them being female and 13 males. The NIST Standard Reference Material for Human Plasma (SRM1950) was purchased from Sigma Aldrich Chemicals Co., (St. Louis, Mo, USA).

For the metabolomics evaluation directly before analysis, the samples were thawed on ice. Then, 75 μL of precooled ISP precipitant solution (ClinMass Steriods in Plasma LC–MS/MS Complete Kit (RECIPE Chemicals + Instruments GmbH, Munich, Germany)) was added to 50 μL of plasma, and the mixture was vortexed for 30 s and left for 10 min before centrifugation at 14,000 rpm for 10 min at 4 °C. The supernatant was separated, and 40 μl of it was subjected to metabolite analysis by SCIEX X500R UPLC − QTOF/MS. The ISP contains six internal standards steroids in its panel, including d7-androstenedione, d4- cortisol, d5- DHEAS, d5-11-deoxycortisol, d8-17-hydroxyprogesterone, and d3-testosterone.

### Instrumentation

HPLC separation was performed using the SCIEX ExionLC AC system on a Phenomenex: Luna Omega 3 m Polar C18 100A column. Data were collected on a SCIEX X500R QTOF mass spectrometer with SCIEX OS software: TOF–MS survey scan with Information Dependent Acquisition (IDA)—triggering of up to 16 product ion scans. Data processing was also performed in SCIEX OS software with simultaneous identification and quantification being accomplished in single software (all devices from AB SCIEX, Framingham, U.S.A. ).

Forty microLiters of the sample was injected for chromatography separation at 30 °C, with a flow rate of 0.8 mL/min, 0.1% formic acid and 2 mM ammonium formate in HPLC water as mobile phase A and HPLC acetonitrile and methanol (50:50) plus 0.1% formic acid and 2 mM ammonium formate as mobile phase B. A 26-min linear gradient was set as follows: 0 min: 2% B, 0–1 min: 2% B, 1–16 min: 98% B, 16–20 min: 98% B. The SCIEX X500R QTOF system with a Turbo V source and capable of electrospray ionization (ESI) was used in positive polarity. The ion source temperature was set to 500 °C, and the ion source voltage was set to 5500 V. An information-dependent acquisition (IDA) method consisting of a TOF–MS survey (250–1100 Da for 350 ms). The declustering potential (DP) was set to 80 V. The collision energy (CE) was set to 10 eV with a collision energy spread (CES) of ± 0 eV. To achieve the most complete MS/MS coverage, the dynamic background subtraction (DBS) function was activated.

### Data processing

MarkerView Software 1.3 (SCIEX) was used to process, align, deconvolute, and normalize (sum of total area) the obtained raw data in which the retention time (RT) was from 0.5 min to 26 min. Mass and RT tolerance values were set to 10 ppm and 0.15 min. Mass and RT of internal standards used for analysis include d7-androstenedione, d4- cortisol, d5- DHEAS, d5-11-deoxycortisol, d8-17-hydroxyprogesterone, and d3-testosterone introduced via the ISP mixture during sample preparation. Principal component analysis (PCA) was used to visualize the system stability of the system and sample distribution. Orthogonal partial least squares discriminant analysis (OPLS-DA) was used to identify the variables responsible for the discrimination. The system stability (RSD %) of m/z, peak areas and retention times were as follows: 0.02 -0.14%, 0.0003–0.0007%, 5.9–8.5%).

A list of the intensities for each detected peak was generated using retention time and the mass-to-charge (m/z) ratio data pairs as the parameters for each ion. Manual scanning of the 10,000 spectral features represented by a unique m/z, retention time, and peak area allowed for generation of a list of 670 peaks of interest used for further evaluation. The Formula Finder algorithm was used to identify potential differential metabolites and generate a group of probable formulas on an unknown ion based on the secondary fragment information, mass error, and isotope distribution patterns.

After data preprocessing, the LipidCreator workbench software in Skyline (available at https://lifs.isas.de/lipidcreator) was used for both quantification and qualification of the lipid species present within the plasma samples^10^. SRM 1950—metabolites by LIPID MAPS consortium, was the basis for comparison and establishment of concentration^19^.

## Supplementary Information

Supplementary Information.

## Data Availability

All data are available in the main text and the supplementary materials. Raw MS data, mzML converted data, interest peak lists, picked and integrated peak areas exported from Skyline and the quantified lipid results tables are available in a public repository (https://dataverse.harvard.edu/dataset.xhtml?persistentId=doi:10.7910/DVN/J6FI2B).
