# Supplementary material for: Inferring a causal relationship between ceramide levels and COVID-19 respiratory distress
Source: Sci Rep. 2021 Oct 21;11:20866. doi: 10.1038/s41598-021-00286-7 (PMC8531370; doi:10.1038/s41598-021-00286-7)
Supplement: Supplementary file 1 — Supplementary Information. [file 41598_2021_286_MOESM1_ESM.docx]

| Title: Inferring a Causal Relationship between Ceramide Levels and COVID-19 Respiratory Distress  **Author:** Mehran M. Khodadoust* |
| --- |

**
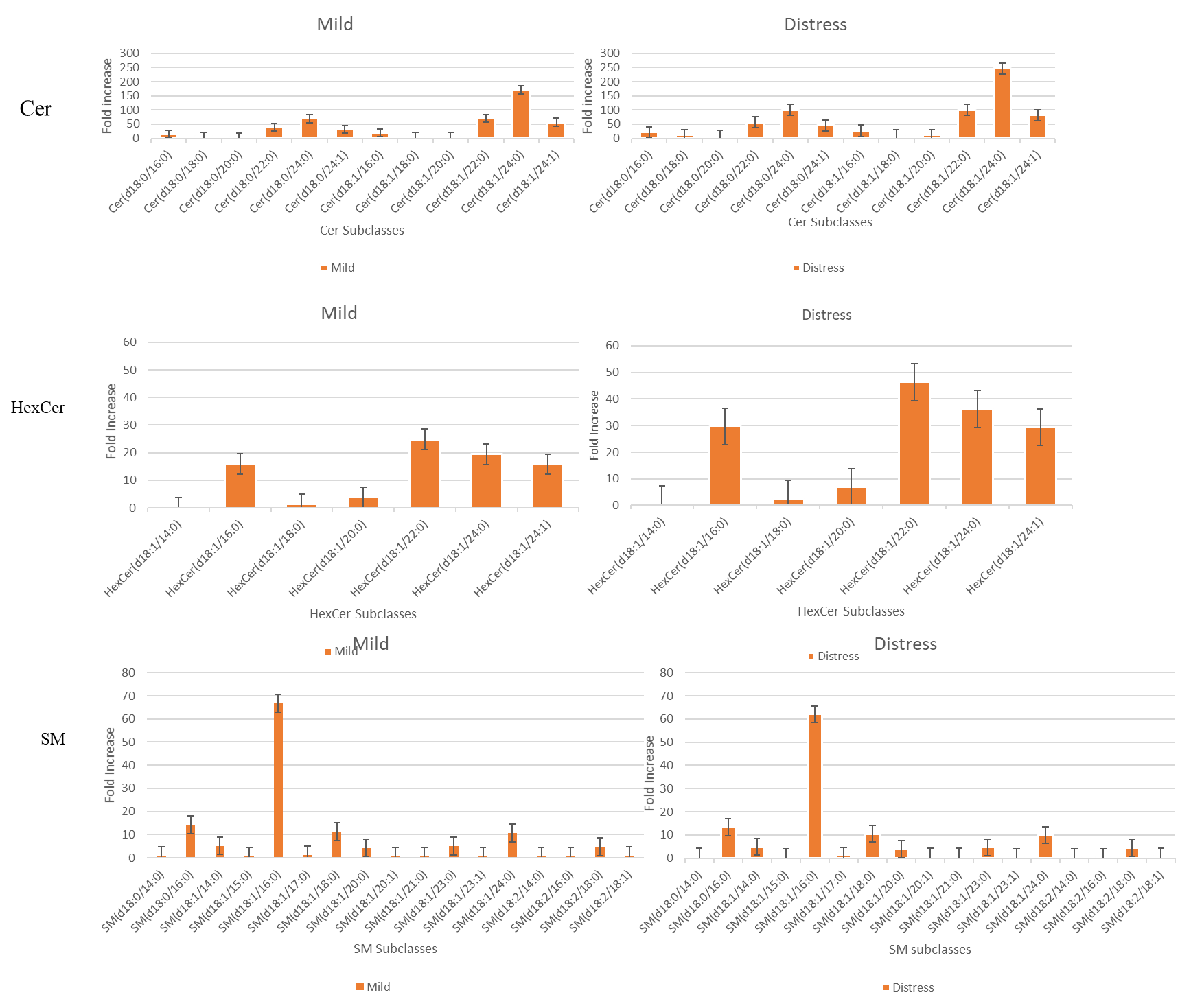
**

**Supplementary Figure 1. Subclass Compositions seen of each lipid class**

Compositional comparison of the subclasses seen in each lipid class between Covid-19 infected with mild vs respiratory distress. Abundance profile of subclasses of ceramides (Cer), Hexosylceramide (HexCer) and Sphingomyelin (SM) in the plasma of COVID-19 infected patients with mild and respiratory distress symptoms in terms of fold changes in observed peak area, compared to the plasma of uninfected individuals. Error bars represent, standard deviation of replicates.


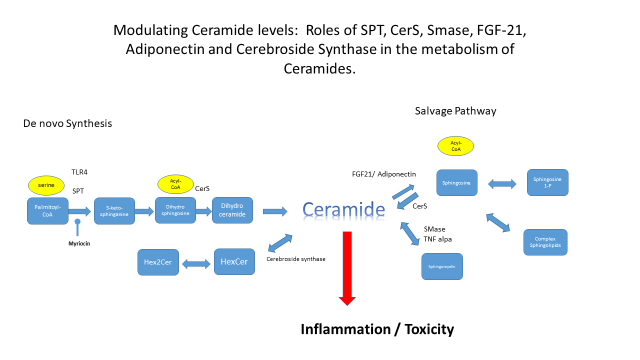


**Supplementary Figure 2. Ways to modulate Cer levels**

Schematic illustration of models for both Cer synthesis and clearing. Cer are synthesized de novo by reaction between Palmitate-CoA and serine mediated by SPT enzyme. The activity of this enzyme can be modified by activation of the TLR4 receptor and the fungal toxin, Myriocin (1). Salvage pathways for synthesis and clearing of Cer’s include the sphingosine, sphingomyelin and Hex ceramides synthesis mechanisms. Factors such as FGF-21 and TNF-alpha that have been shown to mediate activity and regulation of specific enzymes in these pathways (2).

1. Bandet, C. Tan-Chen, S. Bourron, O. Le Stunff, H. Hajduch, E. Sphingolipid Metabolism: New Insight into Ceramide-Induced Lipotoxicity in Muscle Cells. Int J Mol Sci. 2019 Jan 23;20(3):479. doi: 10.3390/ijms20030479. PMID: 30678043; PMCID: PMC6387241.
2. Holland, W. Adams, A. Brozinick, J. et al. An FGF21-adiponectin-ceramide axis controls energy expenditure and insulin action in mice. Cell Metab. 2013 May 7;17(5):790-7. doi: 10.1016/j.cmet.2013.03.019. PMID: 23663742; PMCID: PMC3667496.
